# A biomechanical and histological comparison of the suture bridge and conventional double-row techniques of the repair of full-thickness rotator cuff tears in a rabbit model

**DOI:** 10.1186/s12891-015-0601-7

**Published:** 2015-06-16

**Authors:** Wenyong Fei, Weichun Guo

**Affiliations:** Department of Orthopaedics, Renmin Hospital of Wuhan University, Wuhan, Hubei 430060 China

**Keywords:** Full-thickness, Rotator cuff tear, Suture bridge, Double-row, Rabbit

## Abstract

**Background:**

The suture bridge (SB) technique and conventional double-row (DR) are both effective in repair of full-thickness rotator cuff tears . However, increasing numbers of scholars believe that the SB technique produces better results than conventional DR because of the higher bone-tendon contact area and pressure. However, The clinical outcomes have been mixed and little direct evidence has been supplied in vivo. This study was designed using the SB and DR techniques to determine which is the better technique.

**Methods:**

Sixty-four New Zealand white rabbits were randomly divided into 2 groups, the SB group and DR group. SB and DR were then used to repair their rotator cuff tears. Rabbits were then sacrificed at the 2^nd^, 4^th^, or 8^th^ week after surgery and a histological comparison was made. The biomechanical comparison was made at the 8^th^ week.

**Results:**

The load to failure of the SB group was 134.59 ± 17.69 N at the 8^th^ postoperative week, and that was significantly higher than in the DR group (103.83 ± 6.62, *P* = 0.001), but both repair groups remained lower than in the control group (199.25 ± 14.81). Histological evaluation showed that both the SB and DR groups healed at the bone-tendon interface. But there were subtle differences between the two groups in the structure and morphology of collagen fibers and cartilage cells at bone-tendon interface. In general, the collagen fibers of the SB group were more compact than those of the DR group at all times tested. At the 4^th^ and 8^th^ weeks, the collagen fibers and cartilage cells in the SB group were arranged in a column modality, but those in the DR group were distributed horizontally.

**Conclusion:**

The SB technique facilitated healing more effectively than the conventional DR technique. The difference in morphology of collagen fibers and cartilage cells may be related to the difference in bone-tendon contact pressure.

## Background

Rotator cuff tears are common disease of the shoulder among the elderly. Studies have shown that a full-thickness rotator cuff tear does not heal to the original muscle strength and the strength declines during long term follow-up [1]. There are many ways of repairing full-thickness rotator cuff tears. Cadaver studies have shown that SB technique could increase the bone-tendon contact area and pressure and produce better ultimate failure loads than conventional DR technique in cadaver studies [2–4]. However, Park et al. failed to demonstrate clinical differences between the two techniques [5]. A rabbit animal model of full-thickness rotator cuff tears was here used to evaluate the conventional DR and SB in the repair of such tears. The current hypothesis is that the SB technique would not have superior biomechanical or histological performance than the DR for the repair of full-thickness rotator cuff tears.

## Methods

Animal care and all experiments were performed in adherence to national animal experiment center guidelines and approved by ethics committee of hospital (NO. 20120047). A total of 64 adult male New Zealand white rabbits, aged 12 to 13 months, with a mean body-weight of 2.8 kg (range: 2.5 to 3 kg) were used. The rabbits were kept and fed in a single cage respectively. Animals were observed for one week before surgery to confirm that they were healthy and disease-free. The 64 rabbits were randomly divided into two equal­sized groups. Then DR repair was performed in 32 rabbits and the other 32 rabbits were repaired using SB technique. All repairs were performed on the right shoulder.

### Full-thickness rotator cuff tear model

**Fig. 1:**
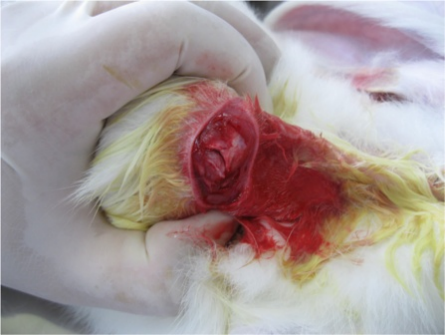
Site of rotator cuff injury—full-thickness tear of the supraspinatus

All operations were performed by the same surgeon with the rabbits under general anesthesia by a qualified veterinary surgeon. Then 0.6 % sodium pentobarbital (4 mg/kg) was injected into the rabbits’ ear veins for general anesthesia. Penicillin (400,000 units) was then injected intraperitoneally to prevent infection. The operation area was sheared and sterilized. Then 2 % lidocaine hydrochloride was injected into the planned skin incision to enhance the effect of anesthesia and reduce post-operative pain. A 3.0 cm longitudinal anterolateral incision was made and the deltoid muscle was exposed. The deltoid muscle was split in the direction of its fibers and the rotator cuff was exposed. The supraspinatus tendon was identified and sharply divided over a 10 mm width, near its insertion at the greater tuberosity. The surface of the greater tuberosity was decorticated using a scalpel to promote healing (Fig. 1).

### Double-row technique for the repair of full-thickness rotator cuff tear

**Fig. 2:**
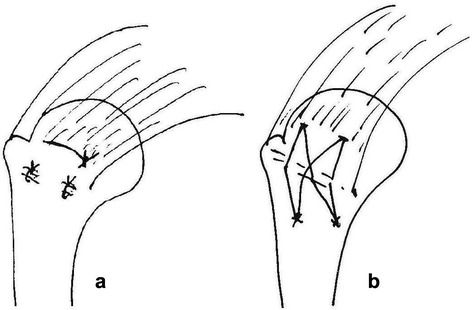
A drawing of the **a**) double-row and **b**) suture bridge repair techniques

The supraspinatus tendon was sutured to the original anatomic insertion using 3.5Metric MERSILK non-absorbable sutures (Johnson and Johnson Co., China). The medial suture was sutured through the greater tuberosity cancellous bone and the supraspinatus tendon at the medial part of the footprint. The second and third sutures were placed equidistant from the lateral part of the footprint in the coronal plane of the humerus head. These sutures were then tied down using a simple suture configuration (Fig. 2a). Hemostasis was examined after the suture, and the wound closed. No restrictions were placed on animal movement. Animals were observed until they were awake and could eat. Every rabbit was kept in a 65 cm × 40 cm × 40 cm cage, and 400,000 units penicillin was injected into the dorsal muscle daily for three days.

### Suture bridge technique for the repair of a full-thickness rotator cuff tear

The supraspinatus tendon was sutured to the original anatomical insertion using the same suture as that used in the DR group. Four sutures were used to make the repairs. Two sutures each were placed through the center of the anterior 1/3 zone and posterior 1/3 zone of the tendon and then through the greater tuberosity of the humeral head in the coronal direction. The other two sutures were oblique and crossed each other (Fig. 2b). Here, oblique sutures were added to increase the contact area and pressure between the supraspinatus tendon and the greater tuberosity. Post-repair treatment of the animals was the same as in the DR group.

### Collection of specimens

Eight of the rabbits in the DR group and eight in the SB group were sacrificed at the 2^nd^, 4^th^, and 8^th^ weeks after surgery for histological assessment. The sixteen rabbits in the two groups were sacrificed at the 8^th^ week postoperatively for biomechanical assessment. At this point, 8 left shoulders of the 16 rabbits were randomly selected as the control group for biomechanical assessment.

The rabbits were euthanized with an intravenous overdose of sodium pentobarbital (100 mg/kg). The supraspinatus muscle with a small part of the scapular blade attached and the proximal half of the humerus were removed en bloc from both shoulders of each rabbit. The longitudinal sections in the center of the repair zone were then prepared. These sections contained the tendon, bone-tendon junction, and greater tuberosity. The left shoulder was also prepared as the control group and compared to the repair specimens. The specimens for biomechanical testing were kept in a box with ice and transferred.

### Histological assessments

The specimens were fixed in 4 % formaldehyde solution for 5 days and decalcified using 7 % nitric acid solution for 1 week. Residual nitrates were removed by immersion in water for 12 h. The specimens were then re-fixed in 4 % formaldehyde solution. After dehydration and embedding in paraffin, 4 μm sections were cut on a rotary microtome, mounted on glass slides and dried overnight at 45 °C. Slides were stained with hematoxylin and eosin and examined under light microscopy. Histological examination was performed by two qualified orthopedic histopathologists in a blinded fashion. Each specimen was thoroughly examined and particular attention was paid to two areas: 1) the bone-tendon repair site where the end of the cut tendon had been attached to the decorticated greater tuberosity; 2) the area of tendon immediately adjacent to the bone-tendon repair. The evaluation focused on the gap between the tendon and bone, the collagen morphology of the tendon, and cartilage growth.

### Biomechanical testing

**Fig. 3:**
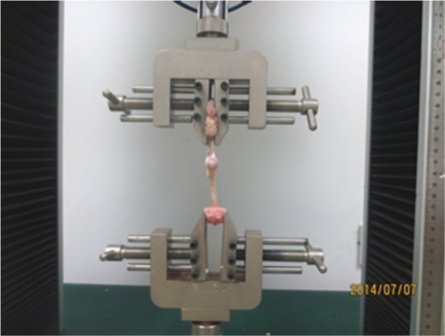
Biomechanical test of load to failure

The biomechanical comparison was assessed at the 8^th^ week after surgery. The specimens were dried with gauze, and the proximal humerus was then potted with dental base acrylic resin powder in a plastic box. The box was removed after the powder solidified. A custom-made clamp was used to grip the solidified body and to mount the experimental preparation to the cross-head of a computer controlled materials testing machine (HY-1080, Shanghai Heng Yi Precision Instruments Co., Ltd.). In order to minimize soft-tissue slippage and failure at the tendon-clamp junction, the proximal part of the supraspinatus muscle-tendon unit was left attached to part of the transferred scapular spine, which was sutured with no. 2, non-absorbable polyester sutures and placed in a modified clamp. The long axis of the tendon and humerus was positioned at an angle of 135° to model the physiological pull of the supraspinatus tendon. Care was taken to ensure equal and symmetrical tension on the tendon before clamping. A cyclic loading test was performed to evaluate the repair of the rotator cuff. A 5 N preload was applied to pre-tension the specimen. The tendon was then cyclically loaded from 5 N to 30 N at 0.1 Hz for 10 cycles. After cyclical loading, each tendon specimen was loaded to failure and the ultimate tensile load was defined as the peak force (Fig. 3).

### Statistical assessment

SPSS 19.0 software for Windows (SPSS Inc., Chicago, IL, U.S.) was used to record the data and for statistical analysis. The effect of intervention (double row, suture bridge or control) was compared for each outcome measure using a One-Way ANOVA statistical test and the Student’s *t* test. A *P*-value of 0.05 was set as the level of significance.

## Results

No rabbits died before they were sacrificed. No rabbits underwent recurrent tearing or wound infection.

### Biomechanical evaluation

Failure of all the remaining specimens occurred at the site of the bone-tendon interface. The weight of rabbits used for biomechanical evaluation and the results of load to failure tests are presented in Table 1. There was no statistically significant difference between the groups with respect to the weight of rabbits (*P* = 0.6, One-Way ANOVA Statistical Test). However, the load to failure of SB group was superior to that of the DR group, and the differences were statistically significant (*P* = 0.001, *T* test). The loads to failure were lower in the DR group and SB group than in the control group, and this difference was also statistically significant (group 1, *P* <0.001; group 2, *P* <0.001, *T* test).

**Table 1:** Weight of rabbits for biomechanical tests and the results of load to failure (mean; SD)

Group	Weight (Kg)	Load to failure (N)
Double-row	3.79 ± 0.12	103.83 ± 6.62
Suture bridge	3.74 ± 0.10	134.59 ± 17.69
Intact control	3.78 ± 0.10	199.25 ± 14.81

### Histological findings

#### Control group

**Fig. 4:**
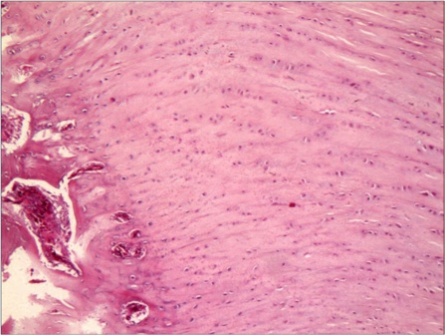
Photomicrograph showing the normal rabbit bone-tendon junction consisting of four different regions: the tendon, unmineralized fibrocartilage, mineralized fibrocartilage and bone. Hematoxylin and eosin, magnification × 20

The normal bone-tendon junction from left shoulder consisted of four different regions: the tendon, unmineralized fibrocartilage, mineralized fibrocartilage, and bone. The most lateral portion, immediately adjacent to the bone-tendon junction, contained longitudinal fibers with some transverse fibers present in layers. This was particularly visible on the medial portion of the bone-tendon junction, where the tendons blended together. Sharpey’s fibers were observed anchoring the tendon to bone and cartilage cells were arranged in columns (Fig. 4).

#### SB group and DR group

**Fig. 5:**
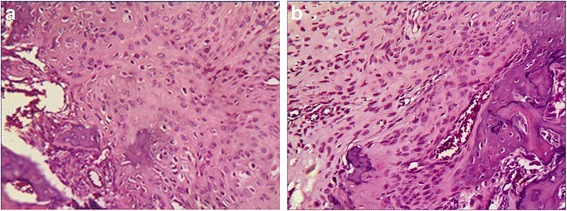
Photomicrographs showing **a** the suture bridge and **b** the double-row technique repair at the2^th^ week. Hematoxylin and eosin, magnification × 20

**Fig. 6:**
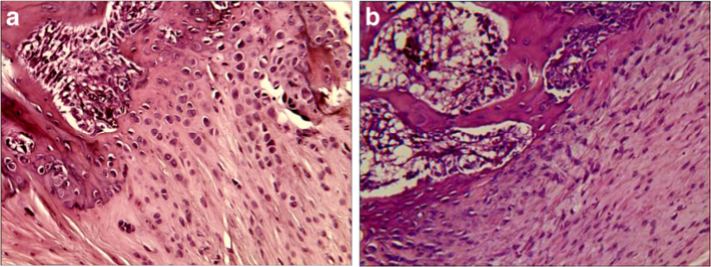
Photomicrographs showing **a** the suture bridge and **b** the double-row technique repair at the4^th^ week. Hematoxylin and eosin, magnification × 20

**Fig. 7:**
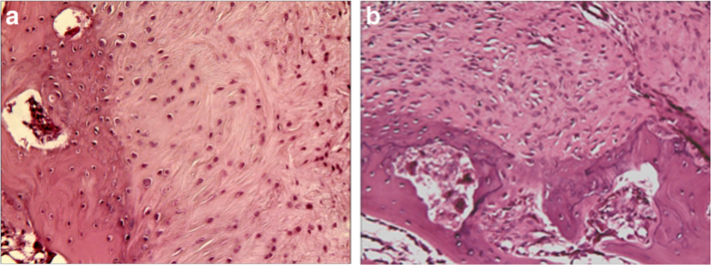
Photomicrographs showing **a** the suture bridge and **b** the double-row technique repair at the8^th^ week. Hematoxylin and eosin, magnification × 20

The stumps of the tendons in both repair groups were closely approximated to the bone at the attachment site at the 8^th^ week postoperatively. No significant gaps formed at the bone-tendon interface during the healing process. However, there were subtle differences between the two specimen groups regarding the growth of collagen and cartilage cells. At the 2^nd^ week after the operation, collagen fibers had attached to the bone in both groups, but those in the SB group were more compact than those in the DR group (Fig. 5a and b). At the 4^th^ week, the collagen fibers in the SB group extended into the cancellous bone naturally with the cartilage cells in columns. Some of the cartilage cells resembled the collagen chondrocytes. However, the collagen fibers and cartilage cells of the DR group were distributed horizontally, and the gradation was significant (Fig. 6a and b). At the 8^th^ week, both groups showed more mature bone-tendon interface structure and morphology than at the 2^nd^ or 4^th^ week. In SB group, the collagen fibers were arranged compactly and in columns that looked like normal structures,anchoring the tendon to bone. However, in the DR group, the collagen bundles were irregular, even those arranged compactly (Fig. 7a and b).

## Discussion

Rotator cuff repair has a high rate of failure. Factors affecting bone-tendon healing after rotator cuff repair include suture strength [6], bone-tendon contact area and pressure [7], fretting between the tendon and bone [8], fatty degeneration of the rotator cuff, the size of the rotator cuff tear [9], the quality of humeral bone [9, 10], suture type [11], and postoperative rehabilitation [12]. Repair techniques may be an important factor affecting the success rate considerably [13]. Numerous biomechanical studies have compared single-row (SR) fixation to DR fixation. Most of these studies have showed that DR repairs reconstruct the anatomic footprint of the rotator cuff significantly better than SR repairs, and DR repairs have less gap formation, better tensile strength, and a lower failure load than SR repairs [14–21]. More and more doctors are selecting anatomic footprint reconstruction, and the suture bridge has recently been described as a new technique because of the increase of the bone-tendon contact area and pressure [22–25]. The contact area of double-row and suture bridge fixation in fresh-frozen human shoulders has been tested previously [2]. Results showed that the mean pressurized contact area between the tendon and insertion was significantly greater for the 4-suture bridge technique (124.2 +/− 16.3 mm^2^, 77.6 % footprint) than the double-row technique (63.3 +/− 28.5 mm^2^, 39.6 % footprint). Their study showed that the SB technique may facilitate healing more effectively than the DR technique. However, a few studies have shown direct evidence that SB fixation has better healing and biomechanical properties in vivo. Clinical outcomes of SB technique have shown mixed results when compared to DR techniques [5, 26–28].

Previous studies using a rabbit subscapularis model have shown that at time zero the SB technique had 38.5 % higher ultimate load than the DR group [29]. A cadaveric study showed the SB technique had a 48 % higher ultimate load than DR repair at time zero [3]. The current study showed that the SB group had a higher ultimate load (134.59 ± 17.69) than the DR group (103.83 ± 6.62) after 8 weeks although both repair groups had significantly less ultimate load than the control group (199.25 ± 14.81) (*P* <0.01). Miyahara et al. studied the mechanical strength of the supraspinatus after reinsertion in dogs and reported that the load at failure was 29.8 % that of controls at two weeks, 62.5 % at six weeks, and 82.5 % at 24 weeks [30], compared to controls [30]. Gerber et al. found that the ultimate failure strength of the reattached infraspinatus in sheep was 30 % at six weeks, 52 % at three months, and 81 % at six months [31]. Their studies showed that solid healing of bone-tendon interface needed more time. This was consistent with the present findings and could explain why the SB group had better healing than the DR group but significantly less load to fail than control group at the 8^th^ week.

Recent reports indicate that the retear rate after surgery remains remarkably high, ranging from 30 % to 94 % [13, 32, 33]. Some clinical follow-up evaluations have reported that the retear rate after a DR repair for large and massive tears ranged from 40 % to 64 % [34, 35]. Mihata et al. compared the retear rates of the DR and SB technique using MRI after arthroscopic rotator cuff repair [26]. The retear rates were 26.1 %, and 4.7 %, respectively, for the DR and SB groups. In the subcategory of large and massive rotator cuff tears, the retear rate in the SB group (3 of 40 shoulders, 7.5 %) was significantly lower than in the DR group (5 of 12 shoulders, 41.7 %, *P* <0.01). The low retear rate indicated that the SB technique could lead to a more robust healing than the DR technique, especially when for large rotator cuff tears. This may have been caused by differences in the healing patterns. At the 2^nd^ week postoperatively, the collagen fibers in SB group were found to be more compact than in the DR group at the bone-tendon junction, and the collagen fibers were arranged irregularly in both groups (Fig. 5a and b). At the 4^th^ week postoperatively, the collagen fibers in SB group extended into the cancellous bone in columns, but those in DR group were distributed indiscriminately with cartilage cells. During this period, the cartilage cells in the SB group also grew in columns containing with collagen fibers, but the morphology of the cartilage cells were immature (Fig. 6a and b). At the 8^th^ week, the structure and morphology of collagen fibers and cartilage cells in SB group were more mature and approximate to a normal structure compared with those in DR group (Fig. 7a and b). Anatomic reduction of fracture could produce a direct healing. For this reason, it was here proposed that the SB repair with more bone-tendon pressure could produce direct healing pattern of bone-tendon interface. The difference in morphology of collagen fibers and cartilage cells at the bone-tendon interface may have been caused by differences in bone-tendon contact pressure.

Currently, there is no clinical evaluation standard including the postoperative retear rate. Park et al. compared the clinical aspects of the DR repair and the SB technique but failed to demonstrate clinical differences between the two techniques [5]. The reason for this may be that the form of clinical evaluation used here is not comprehensive enough. It should include the retear rate. The current study provides direct evidence showing the SB technique to be superior to DR.

There are two limitations to the current study. First, the small area of the rabbit’s greater tuberosity prevented the use of suture anchors, as in human surgery. Similar repairs were made by suturing through the bone. Transosseous suture fixation techniques may produce certain effect to the experimental results. Second, follow-up time was limited; the healing of a rotator cuff needs more time. Some improvements including the repair device and a longer follow-up time should be completed in future studies.

## Conclusion

Previous cadaver studies have shown that SB produces more bone-tendon contact area and pressure than conventional DR technique in cadaver study. The clinical outcomes have been mixed and little direct evidence has been supplied in vivo. The current study showed the healing process and evaluated the efficacy of SB and DR techniques in repairing rotator cuff injuries. The current study showed that the SB technique promoted better healing than the conventional DR technique. The difference in morphology of collagen fibers and cartilage cells may have been caused by the difference in bone-tendon contact pressure.
